# Detection of arbuscular mycorrhizal fungi associated with pecan (*Caryaillinoinensis*) trees by molecular and morphological approaches

**DOI:** 10.3897/mycokeys.42.26118

**Published:** 2018-11-30

**Authors:** L. Fernández idondo, R. P. Colombo, M. Recchi, V. A. Silvani, M. Pérgola, A. Martínez, A. M. Godeas

**Affiliations:** 1 Laboratorio de Microbiología del Suelo. Instituto de Biodiversidad y Biología Experimental Aplicada. Facultad de Ciencias Exactas y Naturales. Universidad de Buenos Aires. Ciudad Universitaria, 4to piso, Pabellón 2. 1428. Buenos Aires. Argentina Universidad de Buenos Aires Buenos Aires Argentina

**Keywords:** *
Carya illinoinensis
*, arbuscular mycorrhizal fungi, spore traits, pyrosequencing, biodiversity.

## Abstract

Arbuscular mycorrhizal (AM) fungal community associated with pecan (*Caryaillinoinensis*) roots and rhizospheric soils was assessed by spore isolation and morphological characterisation and by pyrosequencing of AM molecular markers. The AM fungal community associated with pecan growing in the field, was always more diverse than that associated with pecan growing in containers. This was not observed when AM richness was studied, suggesting that soil disturbance by a reduction in host plant richness leads to a less equitable distribution of AM fungal species, in contrast to natural soils. The chosen primers (AMV4.5F/AMDGR) for pyrosequencing showed high AM fungal specificity. Based on 97% sequence similarity, 49 operational taxonomic units (MOTUs) were obtained and, amongst these, 41 MOTUs corresponded to the *Glomeromycotaphylum*. The number of obtained AM sequences ranged from 2164, associated with field samples, to 5572 obtained from pecan trap pot culture samples, defining 30 and 29 MOTUs, respectively. Richness estimated by conventional species identification was 6 and 9 AM fungal species in soil and pot samples, respectively. *Claroideoglomuslamellosum*, *Funneliformismosseae* and *Entrophosporainfrequens* were the only taxa detected using both techniques. Predominant sequences in the pecan rhizosphere samples, such as *Rhizoglomusirregulare* and other less abundant (*Dominikiairanica*, *Dominikiaindica*, *Sclerocystissinuosa*, *Paraglomuslaccatum*), were detected only by pyrosequencing. Detection of AM fungal species based on spore morphology, in combination with molecular approaches, provides a more comprehensive estimate of fungal community composition.

## Introduction

*Caryaillinoinensis* (Wangenh.) K. Koch (pecan) is considered the most valuable nut-production species of North America and it is currently an expanding crop with great potential. In Argentina, pecan nut production is increasing and has been promoted by many agricultural producers. The existence of thousands of productive hectares between 1 to 50 years old has been estimated (Madero 2013). Since 2005, new suitable cultivars have been introduced, expanding nut production to different geographical regions of Argentina.

Pecan trees have been reported to be associated with ectomycorrhizal (ECM) fungi (Benucci et al. 2012; Bonito et al. 2011; Tarango et al. 2004). However, arbuscular mycorrhizal (AM) fungi were also observed (Brundrett et al. 1990; Muñoz-Márquez et al. 2009; Taber et al. 1982). Mycorrhizal associations in pecan trees may potentially be a key factor for successful acclimatisation of propagated plants in nurseries and in the successful transplanting of trees to field. At both stages, pecan plants are exposed to nutrient and water stressful conditions (Tarango et al. 2004).

The association amongst pecan trees and AM fungal species is still unknown for Argentinian cultivars. Generally, there is limited information concerning the establishment of AM associations with pecan in nature. Conventional (based on spores traits) and molecular methodologies are used for the characterisation of AM fungal communities. It was shown, by molecular analysis, that the ecosystem is more important for the determination of AM fungal community composition than the host species identity (Opik et al. 2006). Moreover, the second-generation sequencing technologies have been increasingly considered as useful tools for identifying AM fungi in environmental samples. In this context, recent metagenomic studies, based on second generation sequencing technologies, have provided new approaches to study microbial community composition in a wide range of environments (Unterseher et al. 2011). Nevertheless, it has been demonstrated that the new sequencing methods can only provide important information when combined with conventional studies on individual microorganisms (Baldrian et al. 2011, Colombo et al. 2014).

The objective of our study was to document the establishment of AM symbiosis in pecan trees growing in a non-native region. Our goal was to characterise, by conventional and molecular species identification, the AM fungal community in soils of an experimental pecan orchard. Complementarily, pecan seedlings were cultured in pots as ‘trap-plants’ to record AM fungal species composition when pecan is the only plant available. Finally, this work seeks to contribute to the existing knowledge of the potential of the pyrosequencing method for the study of AM fungal biodiversity.

## Material and methods

### Sampling and experimental design

Sampling was conducted in the *Estación Experimental Agropecuaria* (EEA) ‘*Delta del Paraná*’ of *Instituto Nacional de Tecnología Agropecuaria* (INTA) in Buenos Aires province, Argentina. Soils of the EEAINTA*Delta* (34°10'S, 58°51'W) are in a deltaic plain with clear fluvial influence where nut production with pecan trees began 40 years ago. The climate is temperate humid with an annual average temperature of 17°C and an annual precipitation of 900–1000mm (INTA 1989). The soil texture was classified as silty clay loam, with pH 4.5, total C 4.48%, organic matter 9.96%, total N 0.3%, extractable P 0.29 meq.100 g^-1^; K 0.47, Ca 9.8, Mg 5.6 and Na 0.47 meq.100 g^-1^. The herbaceous vegetation that appeared under the trees canopy was periodically removed.

In August 2014, twenty pecan trees within the EEA-INTA*Delta del Paraná* were sampled, across an area of approximately 3 ha. Three soil cores of 250 ml (including pecan roots, AM spores and external mycelium) were taken under the canopy of each tree at 20 cm depth, placed in polypropylene bags and stored at 4 °C until processed. All soil cores (sub-samples) from the field were mixed to produce a single complex-sample (T0). This sample was subsequently divided into two parts destined for: i) the molecular and conventional characterisation of AM fungal community; and ii) the establishment of trap cultures in pots with pecan seedlings. Pecan seeds were also collected in the EEA-INTA*Delta del Paraná* during the autumn and stored at 4 °C until use. For stratification, seeds were soaked in tap water, placed in moist perlite and stored at 4 °C for 30 days.

In order to detect the AM fungi colonising pecan roots at sampling time, a microcosm assay was conducted in a greenhouse using the field sample (T0), as AM inoculum. In September, five 10 litre plastic containers were filled with a sterile mix of perlite-vermiculite (1:1) and 400 g of T0. Pecan seeds were surface-disinfected (immersed in 5% sodium hypochlorite solution for 20 min and rinsed with sterile water) and pre-germinated. One seed was placed in each pot. Pecan seedlings were grown under natural light and temperature, watered when necessary and irrigated with 50 ml of Hewitt (1966) nutritive solution without P (to avoid influencing mycorrhization) once a month during 11 months. At the end of the experiment, three sub-samples of the whole root system and rhizospheric soil were removed from each pecan plant and mixed to produce a complex-sample (T1) used for the molecular and morphological characterisation of AM fungal community.

A part of the pecan roots, collected from the field (23 subsamples) and from trap cultures (5 subsamples), were used to evaluate the AM fungal colonisation by observation under a binocular microscope (1000× magnification). Roots were stained by a modified Phillips and Hayman (1970) method: they were bleached with hydrogen peroxide, cleared with KOH (10% w/v, 15 min, 90 °C) and stained with trypan blue in lactic acid (0.02%, 10 min, 90 °C). Intraradical colonisation was quantified by examination of 50 randomly selected root pieces (1 cm length). Frequency (*F*%) of mycorrhizal colonisation was calculated as the percentage of root segments containing hyphae, arbuscules or vesicles (Declerck et al. 2004). Photos were taken under an Olympus BX51 microscope coupled to an Infinity 1 digital camera.

### AM fungal spore isolation and identification

Spores were recovered by successive wet sieving and decanting of soils and collected with a micropipette under a stereomicroscope. In order to ensure that all AM fungal species were sampled, a species accumulation curve was constructed in T0 and T1 as: number of new AM fungal species observed vs. weight of sampled soil. Five grammes of soil were added at each observation opportunity until no new species were found.

Spores characterisation was performed by mounting them in polyvinyl alcohol-lactic acid-glycerol (PVLG) and a mixture of PVLG-Melzer reagent and examining with a binocular microscope (1000× magnification). AM fungi were identified to species, whenever possible, based on morphological characters and subcellular structure of spores with the descriptions available at Professor Blaszkowski web page (http://www.zor.zut.edu.pl/Glomeromycota/) and at Błaszkowski (2012). Taxonomic assignment was performed according to the MycoBank database (http://www.mycobank.org/).

Relative abundance (RA%) of each AM species (calculated as: the number of spores of a particular AM species/the total number of identified AM spores) was plotted in rank-abundance diagrams; AM species were ranked from the most to the least abundant.

### DNA extraction and pyrosequencing

Six metagenomic DNA isolations were carried out from T0 and six from T1 soil samples with the Mo Bio Power Soil DNA isolation kits (Mo Bio Laboratories, INC., Carlsbad, CA, USA) following manufacturer’s protocol. Given the low DNA yields obtained for AM fungi and in order to increase the total AM fungal DNA isolated, all the AM spores and fine pecan roots from 100 g of T0 and 100 g of T1 soil samples (dry weight) were manually collected under a stereomicroscope for DNA isolation with the same commercial kit. All DNA samples from T0 were pooled together as well as those from T1. It has been previously demonstrated that composite samples could provide more accurate surveys than single samples due to the patchy distribution of AM fungi in soils (Xu et al. 2012).

*Glomeromycota* sequences of the small subunits (SSU) region of ribosomal DNA were amplified using the AMV4.5F and AMDGR primers. These primers were chosen because of their high AM fungal specificity (Lin et al. 2012; Lumini et al. 2009) with the aim of quantifying the AM fungal community by amplicon sequencing on a 454 Life Sciences Genome Sequencer FLX System (454 GS FLX) and Titanium chemistry (Roche Applied Science). Oligonucleotides were specifically designed for pyrosequencing with the 454 GS FLX Titanium. Amplicon Fusion Primers contain a directional 454 GS FLX Titanium Primer A or B sequence (in bold letters) which includes a four-base library ‘key’ sequence (underlined) at the 5-prime portion of the oligonucleotide, in addition to the template-specific sequence at the 3-primer end. A Multiplex Identifier (MID) sequence or ‘barcoding’ was added to the reverse primer (in brackets) between the B primer and the template-specific sequences in order to sequence multiple samples in a single run. These sequences allowed automated software identification of each sample. Forward (Primer A – Key):

5’-**CGTATCGCCTCCCTCGCGCCATCAG**AAGCTCGTAGTTGAATTTCG-3’

Reverse (Primer B – Key):

5’-**CTATGCGCCTTGCCAGCCCGCTCAG**(MID10bp)CCCAACTATCCCTATTAATCAT-3’.

PCR amplification was undertaken on a FastStart High Fidelity PCR system (Roche Applied Science, Mannheim, Germany) following the manufacturer’s instructions. The PCR conditions were 95 °C for 5 min, followed by 30 cycles of 95 °C for 45 s, 57 °C for 45 s and 72 °C for 60 s and a final elongation step at 72 °C for 4 min. The reactions were purified and the pyrosequencing run was carried out on one quarter of the sequencing plate on a 454 GS FLX at the Instituto de Agrobiotecnología de Rosario (INDEAR) following the amplicon sequencing protocol provided by the manufacturer.

### Pyrosequencing data analyses

Sequence data were quality controlled and de-noised with the ampliconnoise.py script of Quantitative Insights into Microbial Ecology (QIIME) pipeline (Caporaso et al. 2010). This script also eliminated chimeras. Sequences were clustered into molecular operational taxonomic units (MOTUs) using the pick_MOTUs.py script (QIIME) according to the 97% sequence similarity. This value was chosen in accordance with the conventional definition of microbial species (Konstantinidis et al. 2007). Non-AM fungal sequences were subsequently removed from the data sets (Lekberg et al. 2012) and molecular singletons were not considered in the analyses in order to avoid overestimation of species richness (Unterseher et al. 2011) The most abundant sequences from each MOTU were selected as representative and species identification was assigned comparing MOTUs sequences against the MaarjAM database (http://maarjam.botany.ut.ee/) and the GenBank database (http://www.ncbi.nlm.nih.gov). DNA similarity was performed using BLAST servers and only sequences with coverage and similarity values higher than 98% (resulting in e- values close to zero) were considered. Representative MOTUs sequences, analysed in our study, have been deposited in The Sequence Read Archive (SRA) under the accession number: SRA058132.

Relative abundance (RA%) of each AM MOTUs (calculated as: number of sequences of an AMMOTU/the total number of identified MOTUs) was plotted in rank-abundance diagrams; AM MOTUs were ranked from most to least commonly collected according to their abundance in the samples.

A rarefaction curve was constructed to assess sampling efficiency: the number of new AM fungal MOTUs observed vs. the number of sequences read. To calculate AM molecular richness within T0 and T1 samples, observed species (OS) and Chao 1 richness estimator were performed. Curve analyses were constructed with QIIME by randomly selecting a series of subsets from libraries in different sizes; this procedure was replicated by the programme ten times for each subset sample.

## Results

### Morphological biodiversity of AM fungi associated with pecan trees

**Figure 1.**
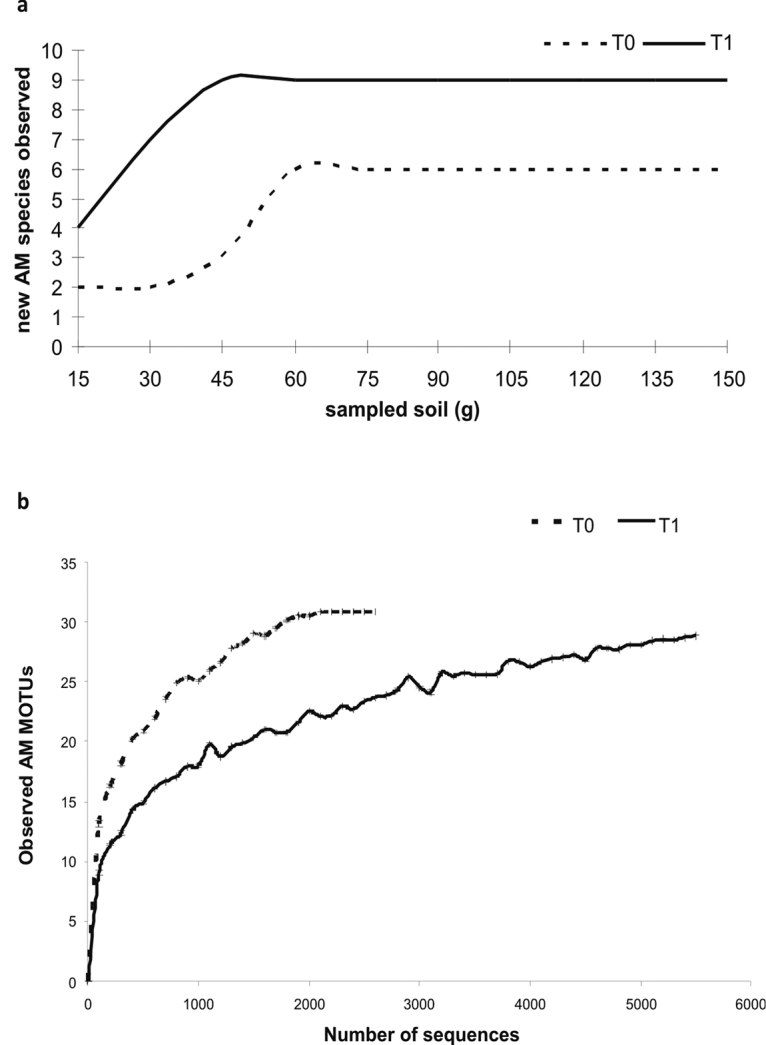
Morphological AM fungal species accumulation curves (**a**) and rarefaction curves (± errors, ten replicates for each subset) of observed AM MOTUs (**b**) detected on *C.illinoinensis* rhizosphere in T0 (field) and T1 (containers) samples.

**Figure 2.**
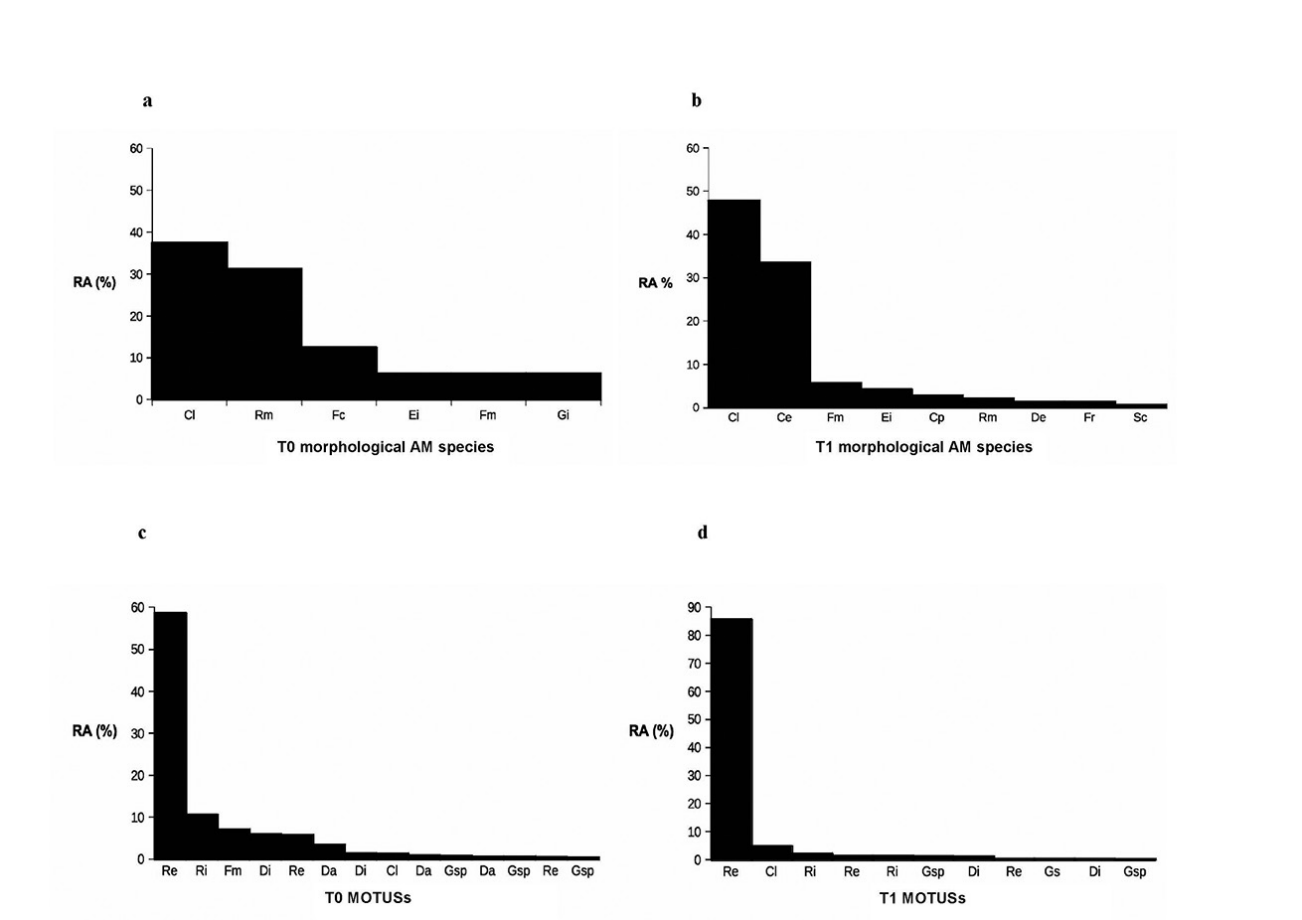
Rank-abundance diagrams of morphological AM fungal species (**a–b**) and rank-abundance diagrams of AM MOTUs (**c–d**) detected on *C.illinoinensis* rhizosphere in T0 (field) and T1 (containers) samples. RA: Relative abundance. Cl: *Claroideoglomuslamellosum*, Rm: *Rhizoglomusmicroaggregatum*, Fc: *Funneliformiscoronatum*, Ei: *Entrophosporainfrequens*, Fm: *Funneliformismosseae*, Gi *Gigasporamargarita*, Ce: *Claroideoglomusetunicatum*, Cp: *Cetrasporapellucida*, De: *Diversisporaeburnea*, Fr: *Fuscutatarubra*, Sc: *Septoglomusconstrictum*, Re: *Rhizoglomusirregulare*, Ri: *Rhizoglomusintraradices*, Di: *Dominikiairanica*, Da: *Dominikiaindica*, Gsp: *Glomus* sp.

After sampling 75 g of both pooled soils (T0 and T1), new AM fungal species were not observed (Fig. 1a) indicating that all AM fungal species were already detected. Based on morphological taxonomic determinations, the AM fungal richness was 6 species in T0 and 9 in T1. Several differences in the AM community structure were found amongst field soil samples and one year old trap pot culture samples: rank-abundance species diagrams (Figs 2a,b) showed that two thirds of the identified spores at T0 belonged to *Claroideoglomuslamellosum* (Dalpé, Koske & Tews) C. Walker & A. Schüssler (37.5% of spores) and *Rhizoglomusmicroaggregatum* (Koske, Gemma & P.D. Olexia) Sieverd., G.A. Silva & Oehl (31%), followed by *Funneliformiscoronatum* (Giovann.) C. Walker & A. Schüssler (12.5%). Spores of *Entrophosporainfrequens* (I.R. Hall) R.N. Ames & R.W. Schneid., *Funneliformismosseae* (T.H. Nicolson & Gerd.) C. Walker & A. Schüssler and *Gigasporamargarita* W.N. Becker & I.R. Hall were also detected (6.25% for each species) in pecan rhizospheric soils. The number of isolated spores at T1 was nearly nine times higher than at T0. In these samples, *C.lamellosum* was also the dominant AM fungal species (49% of spores), followed by *Claroideoglomusetunicatum* (W.N. Becker & Gerd.) C. Walker & A. Schüssler (34%), which was not detected in T0 samples and *F.mosseae* (6%), *E.infrequens*, *Cetrasporapellucida* (T.H. Nicolson & N.C. Schenck) Oehl, F.A. Souza & Sieverd., *R.microaggregatum*, *Diversisporaeburnea* (L.J. Kenn., J.C. Stutz & J.B. Morton) C. Walker & A. Schüssler, *Fuscutatarubra* (Stürmer & J.B. Morton) Oehl, F.A. Souza & Sieverd. and *Septoglomusconstrictum* (Trappe) Sieverd., G.A. Silva & Oehl were also identified with frequencies under 5%.

### Molecular biodiversity of AM fungi associated with pecan trees

Based on 97% sequence similarity, a total of 49 MOTUs were obtained, 41 of them belonging to *Glomeromycotaphylum*, proving the *Glomeromycota* specificity of the selected primers, since the majority of detected sequences corresponded to this phylum (98.5% and 94.8% in T0 and T1, respectively), followed by the *Basidiomycota* fungi *Piriformosporaindica* (0.73% and 4.85% of sequences in T0 and T1, respectively) and *Cyphellopsisanomala* (0.034% of sequences in T1). Sequences of some *Chytridiomycetes* and other *Eukaryota* were also detected (Table 1).

**Table 1.** AM fungal MOTUs and non-target fungi sequence abundance.

Taxonomic assignation	MOTU no.	T0	T1
		Sequence abundance	
** Diversisporales **			
* Entrophospora infrequens *	1	0	2
** Glomerales **			
*Glomus* sp.	2	0	6
*Glomus* sp.	3	4	1
*Glomus* sp.	4	12	0
*Glomus* sp.	5	0	3
*Glomus* sp.	6	2	0
*Glomus* sp.	7	2	0
*Glomus* sp.	8	8	11
*Glomus* sp.	9	0	2
*Glomus* sp.	10	6	66
*Glomus* sp.	11	5	0
*Glomus* sp.	12	2	0
*Glomus* sp.	13	2	0
*Glomus* sp.	14	0	13
*Glomus* sp.	15	0	2
*Glomus* sp.	16	2	0
*Glomus* sp.	17	16	11
*Glomus* sp.	18	0	3
* Claroideoglomus lamellosum *	19	30	272
* Funneliformis mosseae *	20	154	9
* Dominikia indica *	21	75	62
* D. indica *	22	19	16
* D. indica *	23	0	2
* D. indica *	24	2	2
* D. indica *	25	2	0
* D. indica *	26	12	2
* D. indica *	27	2	0
* D. indica *	28	0	2
* Sclerocystis sinuosa *	29	0	2
* S. sinuosa *	30	4	17
* S. sinuosa *	31	0	2
* Rhizoglomus irregulare *	32	1	5
* R. irregulare *	33	11	17
* R. irregulare *	34	125	75
* R. irregulare *	35	1268	4772
* Rhizoglomus intraradices *	36	230	120
* R. intraradices *	37	2	74
* Dominikia iranica *	38	31	0
* D. iranica *	39	129	0
* D. iranica *	40	5	0
** Paraglomerales **			
* Paraglomus laccatum *	41	1	1
Total AM sequences		2164	5572
Total AM MOTUs		30	29
**Non-Target Fungi**			
** Basidiomycota **			
* Piriformospora indica *	42	16	285
* Cyphellopsis anomala *	43	0	2
** Chytridiomycetes **	44	1	10
	45	2	0
	46	0	3
**Other Eukaryota**	47	10	0
	48	2	0
	49	1	4

A total of 2196 and 5876 sequences were obtained for T0 and T1, respectively (there were no chimera sequences). The number of *Glomeromycota* sequences was 7736 (average read length of 268±16bp). Number of sequences ranged from 2164 reads (TO), to 5572 reads (T1), defining 30 and 29 MOTUs, respectively (Table 1). The OS (Fig. 1b) and Chao1 (Data not shown) rarefaction curves reached the plateau phase over 2000 (T0) and 5000 (T1) sampled sequences. The numbers of obtained and estimated MOTUs were close; AM richness appeared to be similar for T0 and T1 with both estimators: 30.8 and 28.9 (OS of T0 and T1) and 37.1 and 35.2 (Chao1 of T0 and T1).

Considering all the *Glomeromycota* genera identified, the proportion of *Rhizoglomus* sequences was always dominant in pecan rhizosphere (83% and 91% in T0 and T1, respectively). *Funneliformis* (7.2% and 0.16% in T0 and T1, respectively) and *Glomus* sequences (8.2% and 4% in T0 and T1, respectively) were more abundant in field samples but clearly diminished in pots. Particularly, *Claroideoglomus* had the opposite behaviour, as their sequences’ abundances were lower at field (1.4%) than at pot (4.9%) level. A few sequences of *Paraglomuslaccatum* (Błaszk.) Renker, Błaszk. & Buscot and *E.infrequens* (less than 0.05%) were also detected in both samples.

The rank-abundance species diagrams (Figs 2c,d) showed that, in T0, almost 60% of the total sequences belong to *Rhizoglomusirregulare* (Błaszk., Wubet, Renker & Buscot) Sieverd., G.A. Silva & Oehl and into the 95% of accumulated sequences, *Rhizoglomusintraradices* (N.C. Schenck & G.S. Sm.) Sieverd., G.A. Silva & Oehl, *F.mosseae*, *Dominikiairanica* (Błaszk., Kovács & Balázs) Błaszk., Chwat & Kovács, *Dominikiaindica* (Błaszk., Wubet & Harikumar) Błaszk., G.S. Silva & Oehl and *C.lamellosum* were also detected. Meanwhile in T1, 90% of total sequences were of *R.irregulare* and *C.lamellosum* was only detected in 95% of the accumulated sequences. The rest of sequences represented only 5%.

Most identified species were common to both soils, some were found only in field samples as *D.iranica*, while others only appeared (*E.infrequens*) or were much more abundant (*Sclerocystissinuosa* Gerd. & B.K. Bakshi) in containers after one year of pecan culture.

### Arbuscular mycorrhizal colonisation of pecan roots

At sampling time, the mycorrhization percentage of T0 natural pecan root was 17% (±6.8), while T1 roots showed 6.33 (±3.53), 28.33 (±15.9) and 29 (±3.6)% of mycorrhization, when observed at 60, 100 and 360 days of seedling growth, respectively.

**Figure 3.**
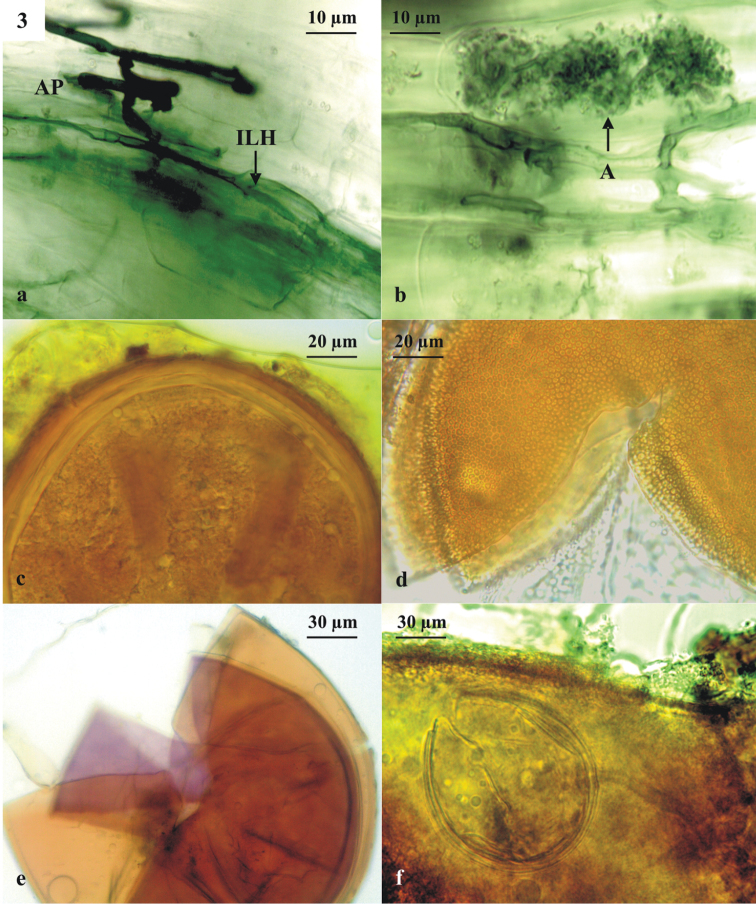
Arbuscular mycorrhizal intraradical colonisation in pecan roots (**a–b**). A: arbuscules, AP: appressoria, ILH: intraradical longitudinal hyphae. Spore of *Claroideoglomuslamellosum* (**c**), *Entrophosporainfrequens* (**d**), *Cetrasporapellucida* (**e**), *Rhizoglomusmicroaggregatum* (**f**) inside another, dead AMF spore, resembling *E.infrequens*.

Appressoria at entry points and intraradical longitudinal hyphae in the outer layers of the pecan root cortex were observed (Fig. 3a). Typical arbuscules were very frequent; however, the presence of vesicles was less commonly detected (Fig. 3b).

## Discussion

Mycorrhization levels observed in pecan roots resemble those reported by Muñoz-Márquez et al. (2009), who registered values of AM fungal colonisation ranging from 13 to 32%. While most forest trees frequently form only one type of mycorrhizal association, ECM or AM (Mosse et al. 1981), some tree species exhibit both symbiosis (dos Santos et al. 2001), suggesting the existence of different niches or soil resources utilisation for these types of fungi in the same root system (Neville et al. 2002). Despite the apparent preference of pecan to associate with ECM in nature, we have demonstrated the AM fungal establishment in pecan roots when growing in natural soils and under greenhouse conditions. These observations are consistent with previous results (Muñoz-Márquez et al. 2009; Taber et al. 1982) reported in areas where pecan is native.

It has been proposed that EM colonisation promotes nutrient mobilisation in organic soils, while AM colonisation enhances the ability to exploit available phosphate in deeper mineral soils (Neville et al. 2002). This dual association in pecan roots could contribute to a better exploration of soil and its consequent geographical expansion to different habitats (Van der Heijden 2001).

Richness of AM fungal molecular species supported by pecan roots was higher in field samples than in pot soils. This was not observed when studying the AM fungal community based on collected spores. This difference between field and pot richness could be explained by the sporulation of some AM species when growing under greenhouse conditions but only found as mycelium in the field. Frequently, soils under different disturbances (such as reductions in plant biodiversity and/or pot effect) are less rich in AM fungal species than natural soils (Fitter 2005; Lumini et al. 2009). This was expected because previous studies demonstrated that the AM fungal community is as diverse as the host plants community (Fitter 2005; Opik et al. 2009). Other causes could be related with the age and density of roots in plastic containers, changes in soil/substrate chemo-physical properties, differences in plant nutritional needs or inherent differences in mature trees compared to seedlings. However, these variables were not analysed in this work.

The representative nature of the different AM fungal species propagules was more even in T0 samples than in T1 (by both methodologies), suggesting a more equitable distribution of richness and a more biodiverse AM fungal community.

AM richness estimated by the molecular approach was between three and five times higher than that estimated by morphological methods. Differences in numbers of morphological species and MOTUs are expected, due to the potential of metagenomic DNA based techniques to detect AM species from all propagules, in addition to spores. It is also possible that overestimation of molecular richness occurs when using a 97% similarity between sequences to define MOTUs (i.e. different MOTUs corresponded with the same AM fungal species). It has been suggested that, with less than 97% of sequence similarity, it might be possible that the ‘query sequence’ and the ’reference sequence’ represent the same taxonomical unit (i.e. fungal species) (Hibbett et al. 2011). Due to the asexual multinucleated nature of AM fungi, a high intraspecific genetic variability within a single individual has been reported (Fitter 2005). We suggest that, when studying this particular fungal group, a lower percent similarity to design MOTUs could give closer richness estimation to the morphological approach.

Moreover, considering that there are a great number of morphologically defined AM fungal species, which are not yet represented in sequence databases, it is very difficult to match AM environmental sequences with those species (Brock et al. 2009).

Considering that mycorrhizal plants richness was very limited in the sampled *Delta* soils, compared with other agro-ecosystems, the AM fungal richness detected in pecan rhizosphere was higher than expected. The comparison of the AM fungal community composition, associated with pecan rhizosphere, indicated that only three species: *C.lamellosum*, *F.mosseae* and *E.infrequens* were detected using both molecular and morphological approaches. The last two were always present at low frequencies, while *C.lamellosum* was the dominant detected species when using morphological technique, but much less frequent in the metagenomic database. Some AM fungal species, such as *F.coronatum*, *G.margarita*, *C.etunicatum*, *C.pellucida*, *D.eburnea*, *F.rubra* and *S.constrictum*, were detected only as spores in soil samples and trap cultures by the conventional approach, but not by pyrosequencing. Considering that not all AM fungal species, defined by the morphological approach, have been sequenced, it is expected that many of the sequences reported here only reached the generic level, or less, when compared with public databases. Moreover, dominant sequences in pecan rhizosphere samples, such as *R.irregulare*, along with other less abundant (*D.iranica*, *D.indica*, *S.sinuosa*, *P.laccatum*), were detected only by pyrosequencing. Spores of these species were not observed, evidencing that these AM fungi were only present as mycelium at sampling time.

Our molecular and morphological data indicated the dominance of *Glomerales* amongst other AM orders. Several genera of *Glomerales* have the ability to extensively colonise roots from spores and mycelial fragments as inoculum source. They already have the ability to form mycelial anastomoses after mechanical disruption. These could be the reasons for their dominance compared to other AM groups. The high frequency of these taxa has been previously observed in natural and agronomic ecosystems by several authors (Dumbrell et al. 2011; Lin et al. 2012; Lumini et al. 2009). An absolute predominance of the *Glomeraceae* spores has also been reported for other trees (Husband et al. 2002; Opik et al. 2006; Wubet et al. 2003).

Results of both approaches were not consistent. Both methods should be integrated to provide a more comprehensive estimate of fungal community organisation. Detection of AM fungal species, based on spore morphology in soil and trap culture, could be used in combination with the molecular approach. More pecan orchards should be sampled to determine the regional distribution of AM associated with these forest crops, even though this first study offers a consistent overview of the AM fungal community present in the rhizosphere of transplanted pecan trees. Given the importance of the correct use of mycorrhizal inoculum (natural or external) to maintain or restore forest populations, knowing the type of mycorrhizal association could be helpful to develop strategies for forest management and reforestation practices. Furthermore, since there are water and nutritional needs inherent to each stage of pecan plants development (nursery growth and field transplant), in future, we aim to study how mycorrhization differentially affects nutrients and water access at each stage.
